# The Multifaceted Roles of PI3Kγ in Hypertension, Vascular Biology, and Inflammation

**DOI:** 10.3390/ijms17111858

**Published:** 2016-11-08

**Authors:** Marialuisa Perrotta, Giuseppe Lembo, Daniela Carnevale

**Affiliations:** 1 Department of Angiocardioneurology and Translational Medicine, IRCCS Neuromed, 86077 Pozzilli, Italy; m.perrotta1@studenti.unimol.it (M.P.); giuseppe.lembo@uniroma1.it (G.L.); 2 Department of Molecular Medicine, Sapienza University of Rome, 00161 Rome, Italy

**Keywords:** PI3Kγ signaling, high blood pressure, immune cells

## Abstract

PI3Kγ is a multifaceted protein, crucially involved in cardiovascular and immune systems. Several studies described the biological and physiological functions of this enzyme in the regulation of cardiovascular system, while others stressed its role in the modulation of immunity. Although PI3Kγ has been historically investigated for its role in leukocytes, the last decade of research also dedicated efforts to explore its functions in the cardiovascular system. In this review, we report an overview recapitulating how PI3Kγ signaling participates in the regulation of vascular functions involved in blood pressure regulation. Moreover, we also summarize the main functions of PI3Kγ in immune responses that could be potentially important in the interaction with the cardiovascular system. Considering that vascular and immune mechanisms are increasingly emerging as intertwining players in hypertension, PI3Kγ could be an intriguing pathway acting on both sides. The availability of specific inhibitors introduces a perspective of further translational research and clinical approaches that could be exploited in hypertension.

## 1. Introduction

**Figure 1:**
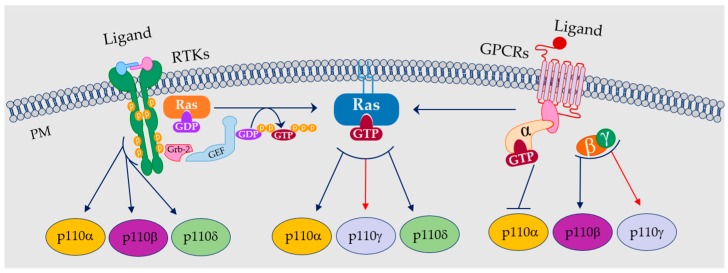
Phosphoinositide 3-kinases (PI3Ks) can be activated upon plasma membrane (PM) receptors, tyrosine kinases receptors (RTKs), and G protein-coupled receptors (GPCRs). p110α, p110β, and p110 bind directly with phosphotyrosine of RTKs (**left**); whereas, p110α, p110β, and p110γ are activated by GPCRs (**right**). Both RTKs and GPCRs also activate Ras, which in turn activates p110α, p110γ, and p110δ.

Phosphoinositide 3-kinases (PI3Ks) are enzymes that mediate intracellular transduction pathways by catalyzing the phosphorylation of phosphatidylinositol lipids at the 3-hydroxyl group of the inositol ring upon several stimuli [1,2]. According to their substrate affinity, they are grouped into three classes (I–III). Class I PI3Ks catalyzes the formation from phosphatidylinositol-4,5-bisphosphate (PI-4,5-P_2_). Class II PI3Ks generates PI-3-P, PI-3,4-P_2_, and PIP_3_, and Class III PI3Ks generates phosphatidylinositol-3-phosphate (PI-3-P) from phosphatidylinositol (PI) [3,4] (Figure 1). Among these, Class I has been extensively characterized for its role in the cardiovascular and immune systems [5,6,7,8].

Belonging to a conserved family, PI3Ks are composed of three domains: the C2 domain, used to bind membranes, the helical domain with a regulatory function, and the catalytic domain with kinase activity [1,2,3,4]. Class I PI3Ks includes two subfamilies with a conserved regulatory subunit but differently activated. Class IA PI3Ks is activated by growth factor receptor tyrosine kinases (RTKs), whereas Class IB PI3Ks is mainly linked to G-protein-coupled receptors (GPCRs) [1,2,3,4]. Class IB PI3Ks has been recognized as a crucial mediator of signaling pathways regulating vascular physiology [4,9,10]. Furthermore, each class is further characterized for comprising different isoforms [1].

While some isoforms of Class IB like p110α and p110β are ubiquitous, p110γ, as well as p110δ, are expressed by specific cells of both cardiovascular and immune systems [1,10,11,12]. In particular, the p110γ isoform has been historically described for regulating leukocytes’ functions [12] and, more specifically, in lymphocytes [12]. Indeed, p110γ isoform contributes to the regulation of certain processes mediating the recruitment of immune cells to inflammatory sites [6] and appears to be involved in pathologies with localized inflammation affecting target organs as kidney and bone marrow [13,14]. Currently, PI3Kγ has been shown to be the principal mediator of effector CD8 T cells migration into target organs [12]. In this review, we will highlight the relevance of PI3Kγ signaling at crossroads between cardiovascular and immune systems. First, we will give an overview of the most significant works published on the role of PI3Kγ pathway in cardiovascular diseases (Table 1). Furthermore, we will describe several inflammatory processes in which PI3Kγ signaling is deeply involved and may be also relevant in hypertension (Table 2).

## 2. PI3Kγ: A Focus on Their Signaling Pathway in Cardiovascular Disease and Hypertension

In the last decade, a consistent piece of literature demonstrated the expression of PI3Kγ in cardiovascular system cells [5,15,16], as vascular smooth muscle cells (VSMCs) [36]. In this regard, it has been shown that PI3Kγ is involved in hypertension [16,17] by regulating vascular function [17] and particularly myogenic tone, defined as the physiological behavior of resistance arteries to counteract perfusion pressure increases and mainly relying on the constrictor tone of VSMCs [17,36,37,38]. Both in experimental animal models of hypertension and in hypertensive patients, it was reported that the increase in peripheral vascular resistances unquestionably contributes to the maintenance of chronic hypertensive status [37,38,39,40,41]. In response to an elevation of intravascular pressure [36,37], the lumen diameter of resistance arteries is subjected to a reduction [36], independently of neural control. Thus, the myogenic response is established as an intrinsic function of VSMCs wall in response to a variety of stimuli [42,43,44] and contributes to the process of blood flow autoregulation in tissues [45]. The myogenic tone is generally described as the sequence of three phases [42]. The first one, also called basal tone, is characterized by an increased intracellular calcium influx through the L-type voltage gated calcium channel (LTCC). The second one consists of a simultaneous higher constriction of VSMC to a steady increase in the intracellular concentration of calcium levels and to a stronger intracellular calcium sensitization. In the last phase, a significant dilation of the arterial wall occurs due to massive calcium entry [46].

To date, research on the mechanisms underlying hypertension has been focused on the involvement of LTCC in VSMCs dysfunction, providing a molecular target to fight the disease [15,16]. It was initially found that PI3Kγ modulates LTCC functions and expression on plasma membranes of VSMCs through Akt signaling [15,16]. Moreover, PI3Kγ signaling has been shown to be necessary in the activation of Ca^2+^ influx in vascular myocytes after stimulation with angiotensin II [47,48]. In past studies, we documented that PI3Kγ, expressed in VSMCs, is crucial to maintaining the balance between vasoconstriction and vasorelaxation induced by GPCRs agonist angiotensin II [17]. We also revealed that mice lacking PI3Kγ are protected from hypertension induced by chronic administration of angiotensin II [16]. Moreover, the absence of PI3Kγ and the expression of a PI3Kγ kinase dead mutant induce a significant reduction of the LTTCs influx and a reduced contractility of vessels upon angiotensin II stimulation [16]. Particularly, we also found that PI3Kγ is able to induce calcium influx through phosphorylation of the LTCC Ca_v_β2a subunit by an Akt consensus site [16]. This finding is strongly correlated with our previous data that demonstrated how the expression of a dominant negative PKB/Akt mutant in vessels reduces the vasoconstriction after angiotensin II exposures [17]. At the same time, both the inhibition of kinase-dependent PI3Kγ signaling and its intracellular signaling mediated by Akt impair vascular myogenic tone [16].

Beyond its relevance in the regulation of angiotensin II-induced L-type-dependent Ca^2+^ influx, PI3Kγ signaling has also been demonstrated as a crucial regulator of oxidative stress in vascular tissues of mice infused with angiotensin II [17]. Considering that myogenic tone and oxidative stress have been historically linked [39], it is conceivable that PI3Kγ plays an additional role in vascular inflammatory responses in hypertension [16]. On this latter note, it is now clearly evident that hypertension is also characterized by immune cells infiltration in peripheral target organs. We and others have observed a significant early infiltration of immune cells in hypertensive mice aorta and kidneys, as well as an enhancement of the costimulation pathway, necessary for the activation and egression of T cells and provided by antigen-presenting cells (APCs) [41,49,50]. Nevertheless, how this process is activated in hypertension remains still partially unfolded. Early studies showed that CD28 binding to B7-1 and B7-2 ligands on APCs mediates costimulation and is activated by PI3Ks [31]. Thus, the isoform p110γ could acquire outstanding relevance in the investigation of molecular mechanisms underlying T cell mobilization in hypertension.

Furthermore, PI3Kγ signaling has also been shown to be involved in other mechanisms related to vascular function, since the activation of Akt can alternatively promote the target blockade through glycogen synthase kinase 3 (GSK-3β) [51]. GSK-3β, in turn, phosphorylates several proteins, maintaining their inactive state and promoting their degradation. Furthermore, Akt is involved in the control of vascular tone through phosphorylating and activating the nitric oxide synthase NOS3 [52], promoting angiogenesis and regulating vascular homeostasis [53]. When its signaling is mediated by phosphatases SHIP1 and SHIP2, as is the Phosphatase and tensin homolog (PTEN), PI3Ks have the opposite action [18,19,54,55]. PTEN mainly has a PDZ domain inhibiting the PI3K-mediated activation of PKB [55].

A last aspect deserving attention is a peculiarity of PI3Kγ that has a dual function [20]: a kinase-dependent activity that positively regulates Akt signaling [16] and a kinase-independent activity that reduces the cAMP levels through PDE3B [20,21,22,23,24]. Recently, we asserted the importance of their kinase-independent activity in the integrity of synaptic plasticity and noradrenergic neurons transmission of locus coeruleus (LC), providing an unpredictable role for PI3Kγ in the neuronal excitability of LC [56]. Changes in PI3Kγ expression and modulations of the downstream pathway have been investigated as potential therapeutic targets in several pathologies affecting the vascular system, the neuronal system, and, more interestingly, immune disorders [9,25,27,57,58].

The genetic ablation of the p110γ subunit or that carry a kinase-death form of PI3Kγ allows for the dissection of molecular mechanisms undergoing immune cell activation in cardiovascular diseases [16,17,27]. In inflamed lesions, p110γ is required for the activation of Akt pathway kinase-dependent in macrophages in response to angiotensin II both in vitro and in vivo, suggesting a possible target to fight atherosclerosis in mice [26,59]. Furthermore, angiotensin II signaling, which promotes oxidative stress and plaque deterioration [59], is markedly reduced in p110γ-null macrophages [20]. In view of these data, the proposal of PI3Kγ as a link between the angiotensin II pathway and the immune responses in atherosclerosis and probably in hypertension is convincing. These data were partially confirmed when it was demonstrated that PI3Kγ signaling was involved in the regulation of vascular myogenic tone [16].

Although our past data have added further knowledge on the mechanisms regulating myogenic tone in resistance arteries, by modifying the LTCCs, it still remains unclear how the activation of immune cells in hypertension induced by angiotensin II is established.

## 3. PI3Kγ in Immunity

**Figure 2:**
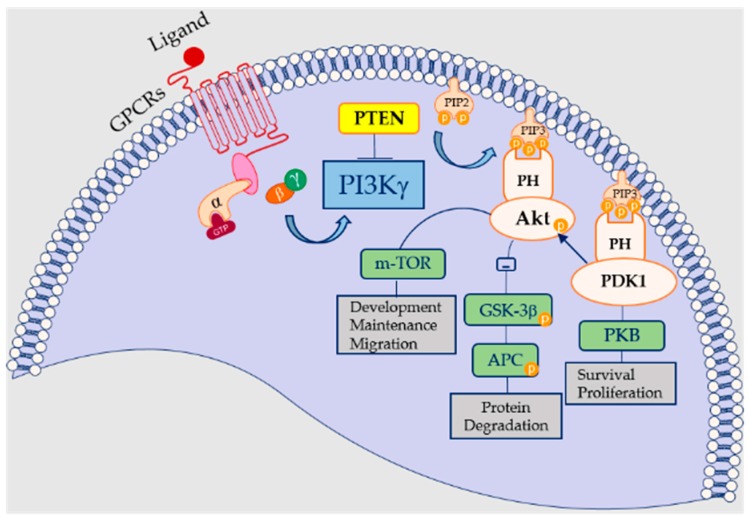
Representation of the main signalings in immune cells. Upon G protein-coupled receptors (GPCRs), PI3Kγ catalyzes the PIP_3_ formation from PIP_2_. PIP_3_ binds target proteins, as Akt and PDK1, with PH domains activating intracellular pathways, as development and maintenance through m-TOR and as survival and proliferation through PKB. PIP_3_ can also mediate protein degradation through GSK-3β signaling. PTEN can inhibit PI3Kγ signaling through PIP_3_ dephosphorylation.

Upon agonists of GPCRs, p110γ-deficient mice display a compromised response of both immune and inflammatory cells [29,60], confirming the crucial role of the p110γ isoform in the activation of those cells participating in a wide variety of inflammatory processes (Figure 2). It was found that mice lacking p110γ show an impaired neutrophil chemotaxis simultaneously to a reduced thymocyte survival and to T cells activation [29,60]. Indeed, the p110γ catalytic subunit is required for a correct T cell development of secondary lymphoid organs [25]. Additionally, in a murine model of septic peritonitis, macrophages from PI3Kγ^−/−^ mice accumulated in inflamed organs worsen the evolution of the disease [60]. In PI3Kγ^−/−^ mice, PIP_3_ is not effectively produced by neutrophils and the downstream pathway is inhibited; thus, motility dysfunction occurs and the inflammatory response is defective [32,60]. Evidence attesting the importance of PI3Kγ in the regulation of immune responses come from studies in mice that have a genetic ablation of PI3Kγ or that carry a kinase-death form of the proteins that are particularly suitable to explore the activation and differentiation of T cells into an activated phenotype. PI3Kγ^KD/KD^ mice display a dysfunction of the complex TCR/CD28-mediated CD4 and CD8 T cells activation and, especially, a deficiency in CD4 T cells differentiation toward activated Th1, Th2, Th17, and Treg [33]. Indeed, PI3Kγ signaling seems to be indispensable for normal T cells functions.

However, neonatal mice lacking the p110γ isoform exhibit a reduced CD4 cell differentiation and an augmented CD8 cell generation, compromising the CD4:CD8 cell ratio [34]. A CD4:CD8 ratio dysfunction could exacerbate an optimal immune response and could hamper a functional thymus growth [35]. In a murine model of inflammatory peritonitis, wild-type mice have a normal migration of naïve T cells, whereas p110γ deficiency mice display a decrease in effector CD8 T cells into inflamed organ, without affecting their proliferation or differentiation [28,32]. Thus, PI3Kγ can differently direct multifaceted roles according to the type of cells in which it is expressed and to the pathophysiological context.

At present, the molecular mechanism of T cells activation in hypertension remains unclear, which makes the possibility of further exploring the role of PI3Kγ signaling in T cells during hypertensive challenges appealing. Indeed, mounting evidence reveals that immune cells are essential to the onset of hypertension induced by angiotensin II or upon DOCA-salt treatment [41,49,61], infiltrating target organs and, more intriguingly, that mice lacking lymphocytes are protected from increased blood pressure [41,49]. More recent data suggest that an oligoclonal population of CD8 T cells is able to decrease the number and size of small blood vessels in the kidney, causing a retention of salt and water and contributing to high blood pressure [62]. The CD86 activation in splenic macrophages, upon angiotensin II, localized in the marginal zone of the spleen, is essential to enhance the T cells egression and consequent infiltration into aorta and kidneys [41,49]. These data suggest that a crosstalk between the innate and adaptive immunity is necessary for the onset of hypertension. Furthermore, CD86 is one of the surface markers of maturation towards APCs, mediating the costimulation pathway [63]. We demonstrated that placental growth factor (PlGF) is a crucial player of this pathway [49], thus suggesting that, even the interaction between this signaling pathway and PI3Kγ could be investigated in search of mechanisms that couple vascular and immune alterations contributing to hypertension.

Thus, in experimental settings of hypertension, it could be particularly interesting to investigate the role of PI3Kγ signaling in regulating the activation of CD8 T cells into effector cells. It is important to notice that more recent data investigated other immune functions of PI3Kγ, revealing the existence of an alternative mechanism involved in adaptive immunity activation and dependent on the expression of PI3Kγ in dendritic cells (DCs). Indeed, it was reported that PI3Kγ is required in DC maturation [30]. Particularly, the Ras-PI3Kγ-Akt-mTOR pathway has been shown to be essential for the development of lung DCs [30], suggesting a specific tissue–organ role for PI3Kγ signaling. This latter report suggests that an even more unpredictable complexity could be explored when approaching PI3Kγ signaling in cardiovascular diseases and, particularly, hypertension, where an interaction between vascular and immune systems emerges as a main regulator of blood pressure increases.

## 4. Conclusions and Perspectives

We have previously found that PI3Kγ is a crucial player in the control of blood pressure levels [16,17,57]. On the one hand, PI3Kγ^−/−^ mice are protected from hypertension by a reduction of peripheral resistances [16], and this effect, in the angiotensin II hypertension model, has been observed simultaneously to reduce inflammatory response in vascular tissues [17]. In this scenario, PI3Kγ could be a possible mediator of the intricate crosstalk that also modulates functions of T cells, which are now well known to play a determinant role in the onset of hypertension and in related target organ damage.

It is demanding to investigate novel roles of PI3Kγ in the immune mechanisms related to hypertension, and it will be instrumental to have a more complete knowledge of how a multifaceted signaling pathway could play different roles in the mechanisms regulating blood pressure.

Furthermore, the development of selective inhibitors could help to elucidate distinct biological processes in which PI3Kγ is involved and to identify potential targets of a wide variety of pathologies affecting the cardiovascular system. In our laboratory, we found that a novel molecule, GE21, blocks the kinase-dependent signaling of PI3Kγ and has an antihypertensive effect in mice [16]. More recently, we have described that GE21 also has a protective functional role in diabetic cardiomyopathy, associated to a decrease in inflammation and fibrosis [27]. GE21 administrated in a dose-dependent manner is able to preserve cardiac function. The possibility to further test the pharmacological inhibition of PI3Kγ signaling to approach immune mechanisms in hypertension is the translational step forward that could contribute to the growing research area for therapeutic applications of PI3Kγ inhibitors [16,27,64].

## Figures and Tables

**Table 1:** PI3Kγ involvement in cardiovascular diseases.

Disease	Functions	References
Hypertension	L-type calcium channels in vascular myocytes	[15,16,17]
Heart Failure	Myocardial contractility; Cardiac remodeling	[18,19,20,21,22,23,24]
Atherosclerosis	Plaque stability	[25,26]
Diabetic Cardiomyopathy	Cardiac remodeling	[27]

**Table 2:** PI3Kγ functions in inflammatory responses

Cell Type	Functions	References
Mast Cells	Hystamine release	[8,12]
Neutrophils	Inflammatory recruitment; chemoattractant-mediated signal transduction	[12,28]
Leukocytes	Inflammatory recruitment	[12]
Thymocyte	Thymocyte development	[29]
Myeloid cells	Osteoclastogenesis; bone homeostasis	[14]
Lung-Specific Dendritic Cell	Development	[30]
Lymphocytes	Inflammatory recruitment	[12]
B cell	T cell activation	[13]
T cell	Activation; migration; differentiation; CD4:CD8 T cells differentiation ratio	[31,32,33,34]
Treg	Activation	[35]
